# Exploratory Studies of Mechanical Properties, Residual Stress, and Grain Evolution at the Local Regions near Pores in Additively Manufactured Metals

**DOI:** 10.3390/ma17133277

**Published:** 2024-07-03

**Authors:** Wei Li, Ning Bian, Kishore Mysore Nagaraja, Xinchang Zhang, Hongbing Lu

**Affiliations:** 1 Department of Mechanical Engineering, The University of Texas at Dallas, Richardson, TX 75080, USA; 2 Materials Science & Engineering Department, Idaho National Laboratory, Idaho Falls, ID 83415, USA

**Keywords:** directed energy deposition, porosity, local mechanical properties, local residual stress, grain structure

## Abstract

Directed energy deposition (DED) is gaining widespread acceptance in various industrial applications since its unique manufacturing features allow the DED to print metallic parts with very complex geometries. However, DED inevitably generates a lot of internal pores which can limit the widespread applications of the DED technique. The current studies on DED porosity are mostly focused on analyzing pores’ bulk-scale influences on mechanical properties and performances. Since DED pores have a micro-scale existence, with dimensions ranging from a few microns to several hundred microns, it is fundamental to explore the pores’ influences on the micro-scale, including local mechanical properties, residual stress, and grains near pores. However, this important research direction has been neglected. The objective of this work is to fill the above gap in DED porosity research and acquire a fundamental understanding of the role of porosity on a microscopic scale. The authors used nanoindentation approaches to investigate internal pores’ effects on mechanical properties and residual stress in local regions surrounding the pores. In addition, the grains near pores were observed through EBSD, and simulated with the Kinetic Monte Carlo model. The research findings can be provided for DED researchers and industrial practitioners as technical guidance. Most importantly, the research results can work as a good reference for tracing the source of bulk-scale mechanical performances and properties of DED parts with internal pores.

## 1. Introduction

**Figure 1:**
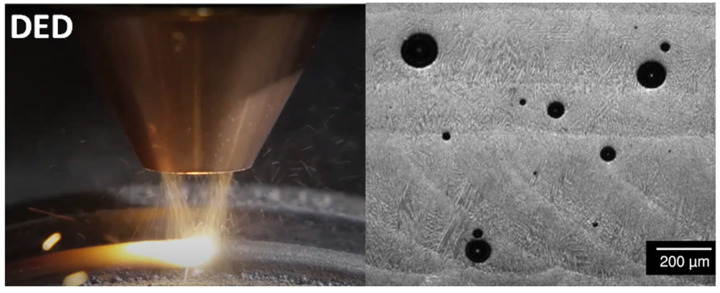
Directed energy deposition (DED) and the pores formed in the printed material [16].

Directed Energy Deposition (DED, Figure 1 [1]), also called laser metal deposition, is gaining widespread acceptance in a variety of applications in the aerospace, automotive, biomedical, and defense industries [2,3,4,5,6]. The unique manufacturing features [7,8,9,10,11] of DED allow it to print metallic parts with complex geometries and structures. The high cooling rate in the DED process yields a large variety of special microstructures whose crystallographic morphologies and fine grains induce enhanced mechanical properties compared to the counterparts made by traditional manufacturing processes. However, DED inevitably generates internal pores with various sizes and shapes, which can limit the wide spread of the DED process for the following reasons [7,12,13,14,15]. The pores in DED-printed parts reduce the load-bearing capability due to reduced load-carrying area and stress concentration around the pores. The pores also induce residual stress and reduce stiffness, which lowers the structural stability. The pores are responsible for significantly lowered fracture roughness and fatigue strengths. In addition, the pores formed in the DED process alter the temperature gradient and affect the cooling rate in the regions near pores, and consequently influence the grain-scale microstructural evolution and morphology.

The pores in DED components can be classified into regular (spherical) and irregular (non-spherical) types according to pore shapes [16]. Small spherical pores (<100 μm) are mainly caused by gas trapped during the powder melting process or the inherent holes in particles formed in the powder synthesis process. The collapse of unstable keyholes in the molten pool flow can form bigger spherical pores (>100 μm), which are sensitively affected by laser energy density. On the other hand, the formation of non-spherical pores is a direct outcome of un-melted powder particles which result from improper DED parameters. Such pores, also known as lack-of-fusion pores, can significantly reduce mass density, facilitate the initiation and propagation of pits, and generate cracks at their corners due to their irregular shapes.

Currently, studies on DED pores are focused on three aspects. The first one is the morphological and spatial characteristics of pores, which are widely studied using both experimental and modeling approaches. Optical microscopy and X-ray Computed Tomography (CT) are often used in experiments to observe the morphology of individual pores (shape and size) and the spatial distribution of a large number of pores. X-ray CT plays a crucial role in the study of the internal structure of additively manufactured porous metals. It provides insights into their internal composition and structural integrity. The X-ray micro-CT allows for high-resolution, three-dimensional imaging that is essential for characterizing the complex geometries and heterogenous structures inherent in the metals. From the micro-CT, detailed information on the pore defect’s size and distribution, connectivity, and phase composition can be critically analyzed. This non-destructive method enables the precise measurement of porosity and its effect on the mechanical properties of the metal structure. This makes X-ray CT indispensable for optimizing manufacturing processes, ensuring quality control, and advancing the development of reliable metal structures. 

With the development of multi-physical modeling approaches, the pores’ morphologies, distribution, and evolution history can be simulated by integrating different models, such as the discrete element method, thermal-fluid model, phase change model, etc. The second aspect of research is focused on the effect of porosity on mechanical properties, an area with increased interest and research activities. A variety of material tests are used to evaluate both the quasi-static mechanical properties (yield and ultimate strengths, ductility, etc.) and the dynamic properties (fatigue life and fatigue strength). The third aspect of research is focused on how to reduce porosity. By adjusting the DED process variables, such as by increasing energy density and decreasing layer thickness, the pore formations are reduced. Some post-process technologies are applied to reduce pore volume and improve the fatigue life of additively manufactured metal parts. Hot isostatic pressing (HIP) is one of such post-process technologies [17,18,19].

Due to the nature of powder-based manufacture, the pores formed in the DED process cannot be eliminated. Therefore, investigating the influence of porosity is of great importance. However, as limited by the capabilities of experimental facilities and the complexity of the modeling, the current studies are confined to the bulk-scale influence of porosity on mechanical performance. Since DED pores are typically micro-scale with dimensions ranging from a few microns to several hundred microns, it is crucial to investigate the roles of porosity at the microscopic scale. However, studies on the influence of DED pores at the micro-scale are extremely rare, and a fundamental understanding is lacking.

The objective of this work is to fill the above gap in DED porosity research and to acquire a fundamental understanding of the influence of porosity at the micro scale. A directed energy deposition machine printed a stainless steel 316L (SS316L) specimen used as a study scenario for this research. X-ray micro-computed tomography (μCT) was carried out to observe and reconstruct the internal pores in the DED-printed metal part. The nanoindentation technique was used to measure the local mechanical properties (hardness, modulus) and residual stress in detail near pores. Electron Back-Scattered Diffraction (EBSD) was applied to observe the grain morphologies near pores. In addition, to investigate the grain evolution with the influence of internal pores, a Kinetic Monte Carlo (KMC) model was used to simulate the grain evolution in the DED process. The spherical pores were initially constructed in the KMC simulation domain.

## 2. Printing the Stainless Steel 316L Specimen

### 2.1. Powder Material

**Figure 2:**
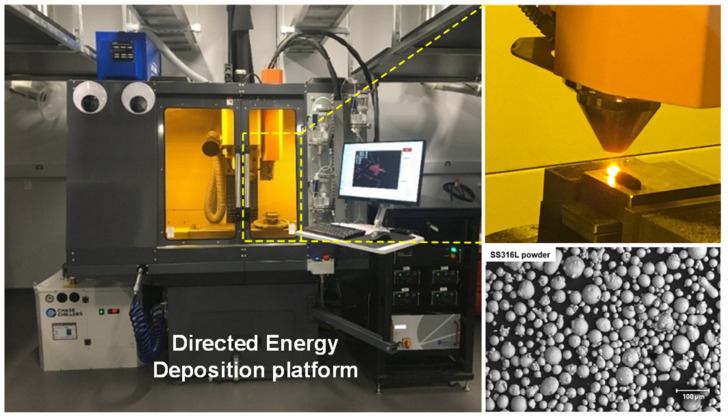
DED platform, DED process, and the SS316L powder used in the study.

The metal powder used in this work was gas-atomized stainless steel 316L (Carpenter Additive, Philadelphia, PA, USA), which had at least 80 wt.% of the particle with a size between 45 and 106 μm, according to the manufacturer datasheet. The SEM images of the metal powder are shown in Figure 2, and their detailed chemical compositions are listed in Table 1.

### 2.2. Directed Energy Deposition

**Figure 3:**
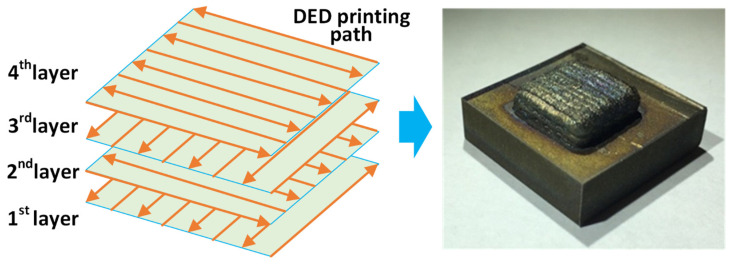
Schematic of the 4-layer SS316L sample with DED printing path and the printed part.

The DED process was performed on a customized DED platform based on a CNC milling machine (Tormach 1100M, Tormach, Madison, WI, USA), integrated with a laser source (Laserline Gmbh, LDM 1000-40, Laserline, Mülheim-Kärlich, Germany) with up to 1 kW power and a powder feeder system (PowderMotionLabs, X2W, Arvada, CO, USA) with four separate chambers. The customized DED platform is shown in Figure 2. The working environment was fully isolated and sealed, with Argon gas continuously filling in to minimize oxidation. The schematic of the DED printing path design and the actual printed specimen are shown in Figure 3. A total of four layers were deposited successively with SS316L powder input. The microscopic image in Figure 2 describes the particle shapes and particle size. 

The processing parameters used in this DED experiment are listed in Table 2. The values of those parameters were primarily determined from a series of trial-and-error process tests to fabricate a squared specimen of good geometrical quality, as shown in Figure 3. Five different laser power values were used to fabricate different layers during the DED experiment, because it was observed from experiments that heat generated from the laser melting process tended to accumulate as layers were deposited from bottom to top, and gradually accumulated heat that had not dissipated in time caused specimen defects, such as excessive build-up in the upper layers, uneven layer height, a rough surface finish, etc. Other parameters, like the power feed rate, the scanning speed, and the z-axis movement that could directly determine the layer thickness and specimen profile, were carefully tested and selected to fabricate the final specimen. The dimensions of the SS316L specimen were designed to be 10 mm long, 10 mm wide, and 4 mm tall, which was achieved by G-code programming of the laser beam movement in both horizontal and vertical directions.

## 3. Porosity Observation with X-ray Micro-Computed Tomography

**Figure 4:**
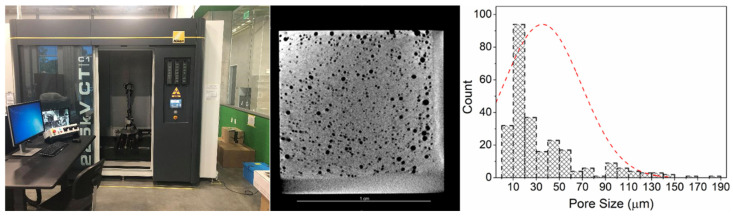
X-ray μCT scan image of a DED-printed sample at a resolution of 18 µm/voxel (scale bar: 1 cm) and pore size distribution.

X-ray micro-computed tomography (μCT) is a non-destructive technique for observing internal pores and cracks in metal AM parts and providing 3D visualization information of these internal features. Currently, a Nikon 225 kV μCT C1 system (Nikon, Tokyo, Japan) with a large workspace (18-inch diameter and 24-inch height) is in use in the authors’ lab. This μCT system can scan a specimen with different dimensions and determine internal features such as pores or cracks inside the specimen accurately. The reconstructed volumetric images can have a resolution of up to 3 μm/voxel. In this work, this X-ray μCT system will be used to non-destructively detect and characterize the internal porosity formed in the DED process, including the pore size spatial distribution, and the volume fraction in the DED specimen. Figure 4 shows a slice of a volumetric image of an L-PBF SS316L specimen revealing the internal porosity using this μCT system. The size distribution of the pores was analyzed based on the scanning observation.

From the CT image, it can be observed that most of the pores in the metal structure were spherical. The pore size can be divided into three categories: micro-pores less than 50 μm, medium pores of 50–100 μm, and large pores of more than 100 μm. Most of the pores in the structure were also micro-pores. The large pores at the periphery of the deposition merged to form an oblong-shaped pore. The effect of these three kinds of pores on the local mechanical properties is further studied using nanoindentation testing. 

## 4. Nanoindentation Experiment for Measuring Local Mechanical Properties near Porosity and the Discussion on the Results

The pores give rise to heterogeneous grain evolution and morphologies, which will likely induce heterogeneous mechanical properties near pore regions. The authors used the nanoindentation experiments to measure the local mechanical properties near pores, including hardness and modulus, based on nanoindentation load-depth curves. In addition, pores formed in DED can complicate the heat transfer process and thermal-mechanical performance, and as a result the distribution of residual stress near pores is likely heterogeneous. To measure the residual stress distributions near pore regions with sufficient resolution, nanoindentation was used as an experimental test to measure residual stress. 

### 4.1. Fundamentals of Mechanical Property Measurement with Nanoindentation Technique

Because of the small size of the pores in a DED-printed component, the testing approach to measuring the local mechanical properties near the pores must be applied to small volumes of materials, which makes nanoindentation a suitable technique. In this work, we applied nanoindentation techniques to characterize the local mechanical properties near pore regions.

The nanoindentation technique provides an effective approach to measuring local heterogeneous mechanical properties of very small volumes of materials [20,21,22], with a load resolution at the µN scale and 0.1 nm resolution in displacement [23,24]. It has been used extensively to measure mechanical properties such as hardness, elastic modulus, contact stiffness, and yield strength that can be extracted from the load–displacement curves obtained directly from an instrumented nanoindentation system [23,25].

**Figure 5:**
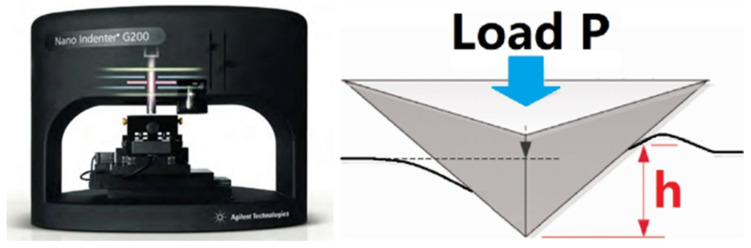
Agilent G200 Nano Indenter system and the nanoindenter illustration.

An Agilent G200 Nano Indenter system (Agilent, Santa Clara, CA, USA) as shown in Figure 5 is available in the authors’ lab. It was used for nanoindentation measurements in this study. This nanoindenter system can reach a maximum indentation depth of 500 μm and a maximum load of 500 mN. The displacement and load resolutions are 0.2 nm and 50 nN, respectively. A range of indenter tips are available, including Berkovich, spherical, punch, and cube corner tips. A diamond Berkovich tip will provide high spatial resolution to measure the mechanical properties. It indents into a flat, polished specimen surface under a constant rate of loading. The applied load on the indenter tip is increased until it reaches a user-defined value. At this point, the load is kept constant and then removed. The load–displacement curve is obtained. Analysis is carried out on the load–displacement curve to determine the mechanical properties of the region near pores based on a contact mechanics analysis of nanoindentation [26,27]. The hardness (H) is obtained using Equation (1), where $P_{max}$ is the maximum indentation force and $A_{c}$ is the contact area corresponding to the contact depth (*h_c_*) at maximum load, and is calculated based on the tip area function. The Young’s modulus $E_{s}$ is obtained using Equation (2), where $E_{r}$ is reduced modulus, $E_{s}$ and $\nu_{s}$ are the Young’s modulus and the Poisson’s ratio of the specimen material, respectively, ande $E_{i}$ and $\nu_{i}$ are the Young’s modulus and Poisson’s ratio of the indenter tip (made of diamond), respectively. Due to the finite stiffness of the indenter tip, its modulus has to be considered in the expression for the calculation of the specimen modulus from the contact stiffness. The contact stiffness (S) is calculated from the slope of the initial unloading curve. Thus, Equations (2) and (3), along with the known values of the area function, slope of the unloading curve, and modulus and Poisson’s ratio values for the indenter tip, can be used to determine the elastic modulus for a specimen corresponding to its Poisson’s ratio.

(1) $$H=\frac{P_{max}}{A_{c}}\;$$

(2) $$\frac{1}{E_{r}}=\frac{1-v_{s}^{2}}{E_{s}}+\frac{1-v_{i}^{2}}{E_{i}}\;$$

(3) $$S=\frac{dP}{dh}=\frac{2}{\sqrt{\pi }}E_{r}\sqrt{A_{c}(h_{c})}\;$$

### 4.2. Local Mechanical Properties near the Single Pore and Pore Cluster

**Figure 6:**
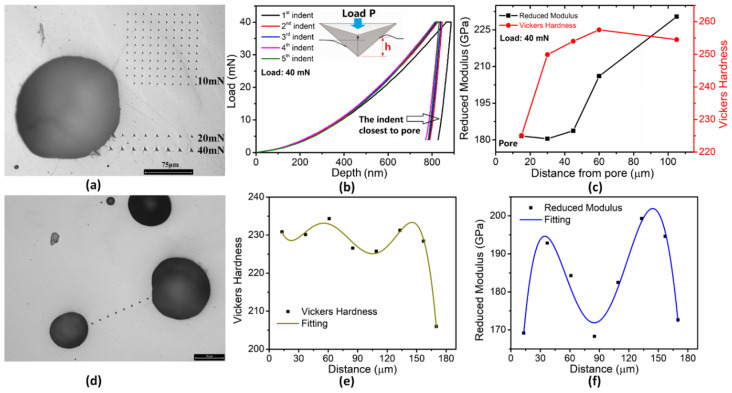
Nanoindentation experimental results in the pore region in a DED specimen: (**a**) optical micrograph of indents near a pore; (**b**) load–depth curves of the first five indents under 40mN load; (**c**) measured elastic modulus and hardness of the first five indents; (**d**) indents between two neighboring pores in pore cluster; (**e**) measured hardness between the two pores; (**f**) measured modules between the two pores.

Nanoindentation tests were performed to measure the hardness and elastic modulus near the pore region in the SS316L DED specimen. The specimen was embedded into graphene powder to form a circular cylinder-shaped nanoindentation specimen under high temperature and high pressure, and it was subsequently polished by coarse- to fine-grit sandpapers to meet the surface requirement for nanoindentation. Then, 10, 20, and 40 mN peak loads were applied on a Berkovich nanoindenter tip to indent the region near the pore at room temperature. The indents are shown in Figure 6a.

The nanoindentation load–displacement curves of the first five indents under 40 mN are shown in Figure 6b. The first indent was the closest to the pore. The closer the distance from the pore, the deeper the nanoindentation depth is. Figure 6c shows that both the elastic modulus and hardness (converted to Vickers hardness value) increase as the position of the indents moves away from the pore. Figure 6d shows the indents between two adjacent pores with different sizes. The hardness near the small pore is higher than the hardness near the big pore (Figure 6e), while the modules are shown to be saddle-shaped between the two pores (Figure 6f).

## 5. Residual Stress Measurement near Porosity with Nanoindentation and Discussion

### 5.1. Fundamentals of Residual Stress Measurement with Nanoindentation Technique

While X-ray diffraction (XRD) allows for the characterizing of residual stress by measuring the change in lattice parameters, XRD has a spatial resolution on the order of millimeters; its low spatial resolution prevents it from measuring the local residual stress occurring in a region on the order of tens of microns (μm) near a pore. Therefore, nanoindentation is a feasible measurement method to measure the residual stress near pores using the theoretical model derived by Suresh and Giannakopoulos [28]. The theoretical approach is described herein. Assume that the residual stress at the surface is uniform over the depth of influence of the nanoindenter tip, and that tensile residual stress can be expressed as a function of hardness H and indentation depth (h and $h_{0}$) in Equation (4), where $\sigma_{t}^{RS}$ is the tensile residual stress, $h_{0}$ is the nanoindentation depth of the reference sample without residual stress, h is the nanoindentation depth of the sample with residual stress, and H is the contact pressure of the indent, also known as the hardness of the reference sample. We used nanoindentation measurements in an annealed specimen as a reference. Compressive residual stress $\sigma_{c}^{RS}$ can be calculated with Equation (5), where an indenter geometric factor, sin *α*, is introduced because the component of residual compressive stress that facilitates contact between the tip and the substrate acts in the normal direction to the inclined face of the nanoindenter tip. For a Berkovich tip, $\alpha =\frac{\pi }{2}-\gamma$, with 2γ being the included angle of the indenter tip, so that α = 24.7° for the Berkovich tip [29,30].

(4) $$\sigma_{t}^{RS}=\left(1-\frac{h_{0}^{2}}{h^{2}}\right)\cdot H\;$$

(5) $$\sigma_{c}^{RS}=\left(\frac{h_{0}^{2}}{h^{2}}-1\right)\cdot \frac{H}{\sin\alpha }$$

### 5.2. Local Residual Stress near a Single Pore and Pore Cluster

**Figure 7:**
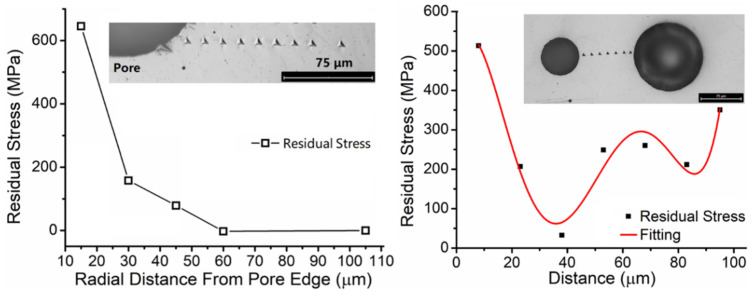
Residual stress near a pore (**left**) and two adjacent pores (**right**) in the sample.

The residual stress distribution near a pore in the DED SS316L specimen (seen in Figure 7) was measured following a series of indents along a line. The hardness values and indent depths from nanoindentation tests are used as inputs to Equations (4) and (5) to calculate the residual stress value at each indent. The residual stress values are plotted in Figure 7 left. It can be seen that residual stress is greater at the indent location closer to the pore. These preliminary results indicate that residual stress distribution concentrates near the pore formed in the DED process. The authors also investigated the residual stress distribution between two neighboring pores with different sizes (Figure 7 right) in the pore cluster. It was found that residual stress distribution follows a reverse saddle shape. Two stress peaks occur close to the two pores’ edges, respectively. A higher stress peak occurs close to the smaller pore edge. Near the middle between two pores, there is another stress peak. These results indicate that residual stress is sensitively affected by the pore distribution in the pore cluster.

## 6. Grain Observation near Porosity and Discussion on the Results

### 6.1. Microstructure Characterization with EBSD

**Figure 8:**
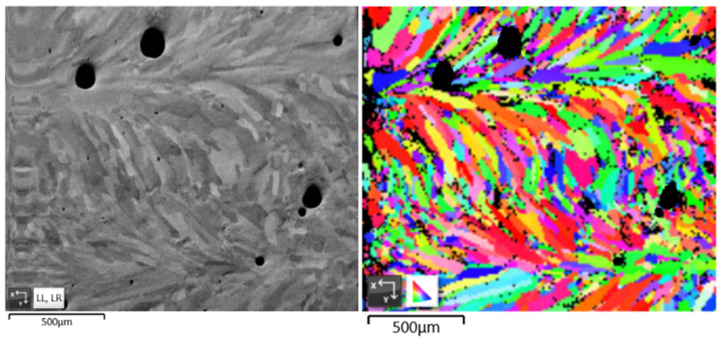
Porosity in the metal sample and grain morphology in the EBSD result.

We observed the grain morphology in the specimen through a series of experimental procedures: sectioning the printed part, mounting in epoxy, grinding, and polishing. After that, we used Electron Backscatter Diffraction (EBSD) to characterize the grain morphology. A Java-based image processing program (ImageJ 1.54j) was used to quantify grain morphology by measuring grain size, shape, and distribution [8,9]. As shown in Figure 8, the DED-printed specimen (SS316L) was observed with EBSD. The columnar grains generated by a high cooling rate are the primary grain morphology. It can be found that some columnar grains are cut off by pores in the EBSD image.

### 6.2. Simulating the Grain Evolution with a Consideration of Internal Pores

Internal pores always form in the molten pool before the solidification starts during the DED process, so pores can act as internal boundaries for the solidification mechanism in the printed part. This unusual cooling mechanism can sensitively affect the grain evolution in the DED process. In this study, we used Kinetic Monte Carlo (KMC) to simulate the grain evolution in the DED process, and especially the material region distributed with porosity.

### 6.3. Fundamentals of the KMC Model

**Figure 9:**
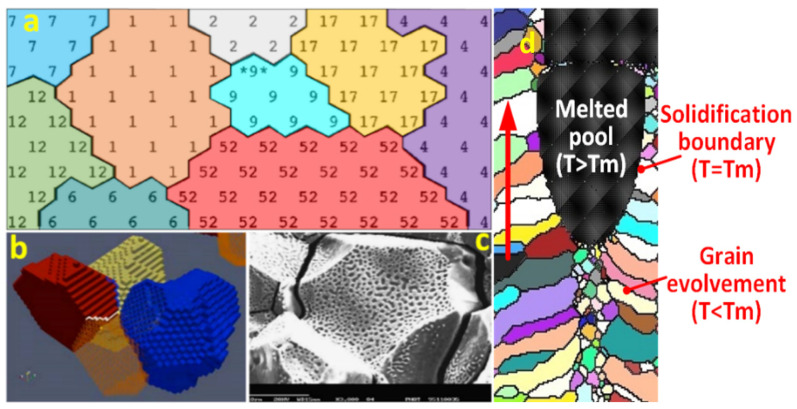
Grain and boundary in KMC model: (**a**) 2-D view of grains with index number; (**b**) 3-D view of grains [33,37]; (**c**) real grains [32]; (**d**) the mechanism of grain evolution. The “*” is just used to indicate a grain randomly selected.

In the KMC Potts model, each lattice site is assigned an integer index, which indicates crystal orientation. The index number (crystal orientation number) ranges between 1 and *n*, where *n* is the total number of possible crystal orientations. The adjacent sites with identical crystal orientation numbers form an individual grain. The interfaces between the nearest neighboring sites with different indices are used to indicate grain boundaries (Figure 9a) [31,32,33]. The grain boundary energy E is determined by Equation (6) [34,35,36,37], where J is a positive constant defining the scale of grain boundary energy, δ is Kronecker’s delta function, $S_{i}$ is the orientation at a randomly selected site *i*, $S_{j}$ are the orientations of its nearest neighbors, and n is total number of nearest neighbor sites. Each pair of nearest neighbors contributes J to the total energy when they have different orientations.

(6) $$E=-J\sum_{j=1}^{n}\left(\delta (S_{i}{,S}_{j})-1\right)$$

(7) $$P_{r}=\left\{\begin{array}{c} M\times \exp\left(\frac{-∆E}{k_{B}T}\right)\;if\;∆E>0\\ \;M\;if∆E\leq 0\; \end{array}\right.\;$$

(8) $$M=M_{0}\exp\left(\frac{-Q}{RT\left(t,i\right)}\right)$$

The nature of grain evolution is grain boundary migration, which will be modeled by randomly selecting a site and altering its orientation to one of its nearest neighbors’ orientations, and judging the system energy variation caused by the attempted reorientation. The probability of accepting the site reorientation, $P_{r}$, is determined by the Metropolis function in Equation (7) [34,35,36,38,39], where ∆*E* is the system energy change; $k_{B}$ is Boltzmann’s constant; and T is the local site temperature. The site reorientation is successful if the energy change is less than zero, which means that there is a decrease in system energy, and therefore grain growth will happen. Then, if the energy change is positive, the probability of grain growth or grain boundary migration will follow the Boltzmann distribution.

Figure 9d shows a mechanism of grain evolution in the DED process simulated by the authors with the KMC-Potts model. A top view of the grain evolution with the molten pool and solidification boundary is shown schematically. In the DED process, heat transfer from the molten pool to the surrounding material generates a heat-affected zone (HAZ) [35,39,40] with a steep thermal gradient. The grain evolution is concurrently controlled by the mobilities of both the molten zone and HAZ. As the molten pool moves forward, the trailing area solidifies and then the temperature gradient causes grains to grow quickly. A grain boundary mobility with the local temperature is defined in Equation (8) [38] to describe the moves of the molten pool and HAZ, where constant Q is the activation energy for grain boundary motion; $M_{0}$ is the Arrhenius pre-factor; R is the gas constant; and $T\left(t,i\right)$ is the local temperature at time *t* and location *i*. M represents grain boundary mobility. This parameter is crucial in determining how easily grain boundaries can move under the influence of temperature and stored energy in the material. It is temperature-dependent and increases at higher temperatures, which leads to more rapid grain boundary movement. The grain growth rate is usually proportional to the grain boundary mobility. Faster grain growth leads to larger grain sizes over the same period. According to the KMC–Potts model, grains grow by undergoing Monte Carlo switches, unlike indexes of neighboring grains. Index reassignment can take place with indices of formerly solidified grains (causing grain growth), or among lattice sites that just exited the molten pool (causing grain nucleation).

### 6.4. Simulating Grain Evolution Considering the Influences of Porosity

To simulate the grain evolution at the regions near pores, the pore geometries were constructed in the simulation domain. In this modeling work, the porosity region was defined as void through Boolean algebra “NOT”. The external surfaces of pores will be defined as boundary conditions in the simulation because pores are formed during the molten pool evolution and before the grain evolution. The authors used sphere geometry to reconstruct the pores in the KMC modeling domain, and then the KMC Potts model was used to simulate the grain evolution. The molten pool size was acquired from the in situ camera monitor in the DED platform. The DED processing variables (laser scanning speed, hatch spacing, layer thickness, scan pattern, etc.) were input to the KMC model to simulate the grain evolution. 

**Figure 10:**
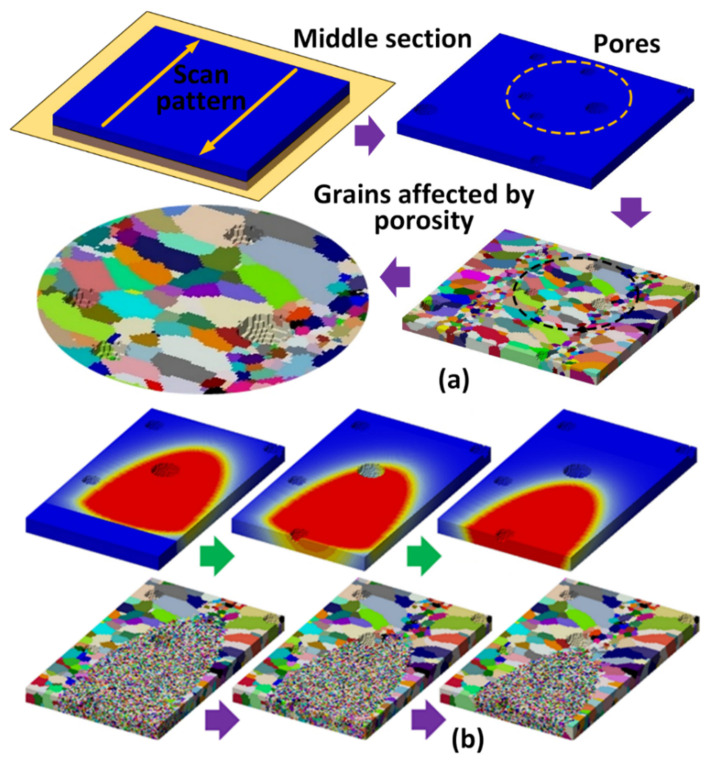
(**a**) The scanning path of the DED process, the porosity constructed in the KMC model, and the simulated grain morphology in the region near pores. (**b**) The grain evolution is simulated by the KMC model. As the molten pool moves forward, the trailing area causes the grains to grow. The porosity structure in the simulation domain can influence the final grain morphology.

Figure 10 demonstrates the intricate relation between porosity and grain morphology in the DED process. The KMC model provides insights into how pores can alter grain growth and enhance microstructural heterogeneity and thus influence mechanical properties. Figure 10a shows a simulated DED process where pores are depicted and included in the KMC model. The scanning path of the DED process follows a zig-zag pattern. It was chosen to create a thermal profile that affects how grains grow and solidify. Further, in contrast, below this, the grain size and structure between pore-adjacent areas and pore-free regions can be observed. The simulation captures how grains evolve as the molten pool moves forward. The molten pool is depicted in Figure 10b, top row, with the red color. The color represents the meltpool region with a temperature higher than the liquidus temperature of the metal. The blue region represents the room temperature of 298 K (24.85 °C). Just below these contours, as this molten pool moves, the grain grows in the trailing and surrounding area. The KMC simulation resulted in columnar grains due to directional solidification. Columnar grains are the primary grain morphology formed in DED and most other metal AM processes. Further, the pores complicate the local grain evolution process. This leads to increased heterogeneity in the grain morphology. Grains tend to be smaller near pores due to the obstruction of grain boundary movement. They could also be affected due to localized cooling effects.

## 7. Conclusions

Since metal DED pores are typically microscopic, with dimensions ranging from a few microns to several hundred microns, it is crucial to investigate the roles of porosity at the microscopic scale. The conclusions of this study are stated as follows. (1) Both hardness and modulus were reduced at the region close to the single pore. Therefore, it can be concluded that the presence of porosity can weaken the local mechanical properties near pores. Pores in metal AM parts usually appear as pore clusters. The distribution of mechanical properties is influenced by the pore cluster. (2) There are concentrations of residual stress (RS) near pores. The pore cluster can complicate the RS concentration. (3) Columnar grains are the primary grain morphology formed in DED. The pores complicate the local grain evolution, enhance the heterogeneity of grain morphology, and reduce the grain size near pores.

By understanding the micro-scale effects of porosity, it is possible to develop strategies to mitigate these effects, leading to improved mechanical properties and performance of DED-manufactured parts. This study addresses a critical gap in the DED porosity research on the micro-scale effects of internal pores. The combination of nanoindentation, EBSD, and Kinetic Monte Carlo simulation provides a comprehensive understanding of how pores impact local mechanical properties, residual stress, and grain behavior, ultimately contributing to the advancement of DED technology. 

However, this exploratory study should be further expanded upon in the future. Based on the 3D view of the internal pore structure, more information on the pore distribution, connectivity, and size should be studied. These studies could provide valuable insight into the dynamics of pore formation, and their correlation with process parameters can be obtained based on in situ monitoring. Further dynamics grain growth modeling to simulate grain growth and recrystallization during the DED process can help to predict and control the grain structure in the deposited structure. Finally, the current research does not include porosity alleviation strategies. The effect of postprocessing techniques such as heat treatment, hot isostatic pressing, ultrasonic nanocrystal surface modification, and laser shock peening to reduce or eliminate pores would be beneficial.

## Figures and Tables

**Table 1:** Chemical compositions of the SS316L powder (wt.%).

Material	Fe	Cr	Ni	Mn	Mo	Nb + Ta	N	Si	C	P	Co	Cu	Ti	S
SS316L	Bal.	16.00–18.00	10.00–14.00	2.00	2.00–3.00	-	0.10	1.00	0.03	0.05	-	-	-	0.03

**Table 2:** The processing parameters in the DED experiment.

Parameter	Value
Laser power	900 W—Layer 1
	600 W—Layer 2
	400 W—Layer 3
	200 W—Layer 4
Power feed rate	4 L/min
Scanning speed	50.8 mm/min
Layer thickness	1 mm
Argon gas flow rate	5 L/min

## Data Availability

Data are contained within the article.
